# Persicaline, A New Antioxidant Sulphur-Containing Imidazoline Alkaloid from *Salvadora persica* Roots

**DOI:** 10.3390/molecules23020483

**Published:** 2018-02-23

**Authors:** Mohamed Farag, Wael M. Abdel-Mageed, Omer Basudan, Ali El-Gamal

**Affiliations:** 1 Pharmacognosy Department, College of Pharmacy, King Saud University, P.O. Box 2457, Riyadh 11451, Saudi Arabia; dr.farag@gmail.com (M.F.); wabdelmageed@ksu.edu.sa (W.M.A.-M.); basudan@ksu.edu.sa (O.B.); 2 Pharmacognosy Department, Faculty of Pharmacy, Assiut University, Assiut P.O. Box 71526, Egypt; 3 Pharmacognosy Department, Faculty of Pharmacy, Mansoura University, El-Mansoura P.O. Box 35516, Egypt

**Keywords:** *Salvadora persica*, persicaline, imidazoline alkaloids, sulphur-containing compounds, radical scavenging activity

## Abstract

*Salvadora persica* L. is a popular chewing stick commonly known as “miswak”. During our ongoing research activities on the chemical constituents of *Salvadora persica* roots, which is a new sulphur-containing imidazoline alkaloid 1,3-Dibenzyl-4-(1,2,3,4-tetrahydroxy-butyl)-1,3-dihydro-imidazole-2-thione, persicaline, (**1**) along with five known compounds (**2**–**6**) are identified. Compounds (**2**, **3**) were reported for the first time from the family *Salvadoraeceae*. The structure of the new compound was established by extensive spectroscopic data and HR-MS. The antioxidant activities of the fractions and isolates were evaluated using different in vitro methods, such as DPPH, superoxide anion and nitric oxide radicals scavenging assays. Compound (**1**) showed a promising antioxidant activity with IC_50_ 0.1, 0.08, and 0.09 µM in the three assays, respectively, comparable to ascorbic acid.

## 1. Introduction

*Salvadora persica* L. (*Salvadoraeceae*), commonly known as meswak, araak, and pilu, is one of the most popular medicinal plants among Muslim population [1]. It is used for centuries as a natural toothbrush and has been recommended by the World Health Organization (WHO) for oral hygiene [2]. From the phytochemical point of view, the roots are rich source of elemental sulphur, salvadourea, *m*-anisic acid, and benzyl isothiocyanate, which has antiviral activity against Herpes simplex virus-1 that affects oral cavity [3]. Moreover, roots have a high content of alkaloids, such as salvadorine, trimethylamine, chlorides, fluorides with small quantities of tannins, saponins, flavonoids, and sterols [4,5,6]. Chemical investigation of *S. persica* also indicates the presence of nitrogen-containing compounds in the sticks, such as pyrrolidine, pyrrole, and piperidine derivatives [7]. Four lignin glycosides were also isolated from the stems as salvadoside, salvadoraside, syringin, and liriodendrin [8]. Recently, a study in 2017 revealed 5-*O*-caffeoylquinic acid and 4,5-*O*-Dcaffeoylquinic acid as the major phenolic compounds in the root while the stem was rich in 5-*O*-caffeoylquinic acid, 3,5-*O*-Dcaffeoylquinic acid, catechin, and epicatechin [9]. A high content of 5-*O*-caffeoylquinic acid, naringenine, and some alkaloids, including caffeine, theobromine, and trigonelline were also reported from the bark [9]. Furthermore, a recent study by Farag et al. compared the chemical composition of three *S. persica* root and one stem samples from different geographical origins (Saudi Arabia and Egypt) [10]. The roots contained more amino acids (2–12%) while stem (1%), with l-alanine (1–10%) being the major one although other amino acids were found at comparable levels [10]. Total nitrogenous compounds were also found at higher amount in roots ranging from 3.2–5% as compared to those of stem (2.86%). Among them, *N*-benzylamine is mostly present in the root while urea in stem. Variations in the chemical constituents among the three root samples (two from Saudi Arabia and one from Egypt) were noticed, which is an evidence of differences due to geographical regions [10]. Four new steroidal esters were recently isolated from the roots as β-sitosteryl arabinosyl vanilloyl stearate, β-Sitosteryl-3-vanilloyl-4′-palmitate, β-sitosteryl-3-vanilloyl-4′-stearate, β-sitosteryl vanilloyl oleate [11]. Previous studies also reported various biological activities, such as antimicrobial, analgesic, anti-inflammatory, anthelmintic, antidiabetic, aphrodisiac, stomachic, antiulcer, anticonvulsant, sedative, hypolipidemic, antiosteoporosis, and antitumor activities [7,8,9,10,11,12,13]. The green synthesis of nanoparticles has been recorded as the latest application of *S. persica* in which its extracts were used as natural reducing agents [7]. 

**Figure 1:**
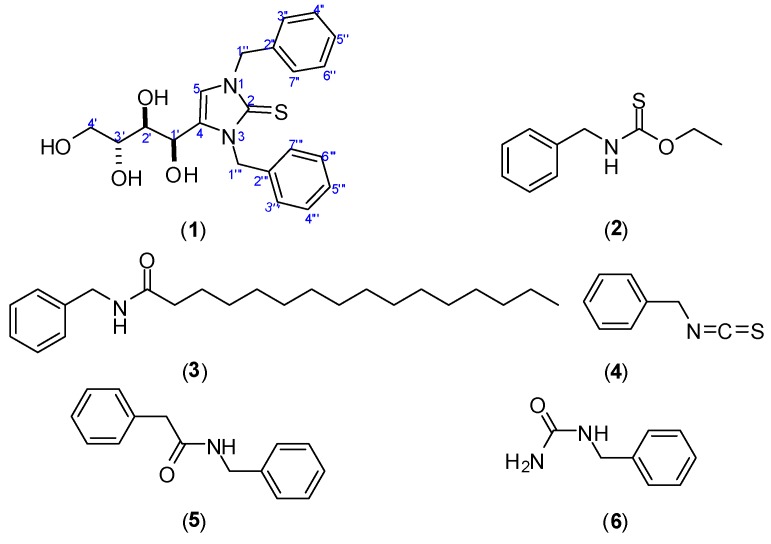
Chemical structures of **1**–**6**.

During the course of our ongoing research activities regarding the isolation and identification of drug leads from natural sources, we had the opportunity to investigate the roots of *S. persica* to identify its constituents and investigate their potential biological activities. The phytochemical study of ethanolic extract of the roots of *S. persica* led to isolation of a new sulphur-containing imidazoline alkaloid “persicaline” (**1**), along with five known compounds (**2**–**6**) (Figure 1). Benzyl-thiocarbamicacid-*O*-ethyl ester (**2**) and hexadecanoic acid benzylamide (**3**) were reported here for the first time from family *Salvadoraeceae,* whilst *N*-benzyl- 2-phenylacetamide (**5**) and benzylurea (**6**) were identified for the first time from the roots [14].

Herein, the NMR data of the new compound (**1**) were reported and the in vitro antioxidant activities were evaluated for the total alcoholic extract, petroleum ether, non-basic, alkaloid-rich fractions, and isolates (**1**–**6**).

## 2. Results

### 2.1. Isolation, and Sructure Elucidation

The dried alcoholic extract was subjected to solvent fractionation and acid base treatment for alkaloid extraction [15]. A combination of normal and reversed phase column chromatography afforded six compounds (**1**–**6**, Figure 1).

Compound **1** was obtained as a light yellow amorphous powder, (${\left[\alpha \right]}_{D}^{25}$ = −4.5°, c 1.08, MeOH). HREIMS showed a molecular ion peak at *m/z* 400.1450 [M]^+^ (calculated for C_21_H_24_N_2_O_4_S, 400.1457), consistent with a molecular formula of C_21_H_24_N_2_O_4_S, thus implying 11 degrees of unsaturation. The presence of sulphur atom is indicated by the presence of isotopic peak (402.1464) [M + 2]^+^. The UV: λ_max_^MeOH^: 208, 271 nm. The FT-IR spectrum (KBr) showed presence of hydroxyl groups (3446 cm^−1^) and aromatic rings (1560, 1533 cm^−1^) in addition to characteristic absorption bands at (1463, 1413, 1270, 1230, 1122, 1029, 730 cm^−1^) attributed to [(-N-C=S) stretching and bending] [16,17]. 

The ^1^H, ^13^C and DEPT-135NMR spectra (Table 1) in combination with 2D ^1^H-^13^C HSQC of (**1**) in DMSO-*d*_6_ (Supplementary data, S2–S9) indicated the presence of three sp^3^ methylene groups H-4′ [δ_H_ 3.53 (1H, m) and δ_H_ 3.37 (1H, m); δ_C_ 63.3], H-1′′ [δ_H_ 5.32 (1H, d, *J* = 15.9 Hz) and δ_H_ 5.23 (1H, d, *J* = 15.9 Hz); δ_C_ 49.9] and H-1′′′ [δ_H_ 5.42 (2H, s); δ_C_ 47.6]; three oxygen-bearing sp^3^methines H-1′ [δ_H_ 4.62 (1H, m); δ_C_ 62.9], H-2′ [δ_H_ 3.38 (1H, m); δ_C_ 71.9] and H-3′ [δ_H_ 3.40 (1H, m); δ_C_ 70.7]; one sp^2^ olefinicmethine H-5 [δ_H_ 7.08 (1H, s); δ_C_ 115.8] and ten sp^2^ aromatic methines grouped by the ^1^H-^1^H COSY experiment into two spin systems of two mono-substituted aromatic systems including H-3′′/7′′ [δ_H_ 7.36 (2H, d, *J* = 7.5 Hz); δ_C_ 127.9], H-4′′/6′′ [δ_H_ 7.31 (2H, d, *J* = 7.5 Hz); δ_C_ 128.4], H-5′′ [δ_H_ 7.31 (1H, m); δ_C_ 127.7]; and H-3′′′/7′′′ [δ_H_ 7.22 (2H, d, *J* = 7.5 Hz); δ_C_ 126.8], H-4′′′/6′′′ [δ_H_ 7.37 (2H, d, *J* = 7.5 Hz); δ_C_ 128.6], H-5′′′ [δ_H_ 7.25 (1H, m); δ_C_ 127.2]. Four hydrogen resonances lacked correlations in the HSQC spectrum of **1** and were therefore recognized as being located on hetero atoms that were identified later as hydroxyl protons for OH-1′ [δ_H_ 5.11 (1H, d, *J* = 8.1 Hz)], OH-2′ [δ_H_ 4.68 (1H, d, *J* = 8.1 Hz)], OH-3′ [δ_H_ 4.55 (1H, d, *J* = 8.1 Hz)] and OH-4′ [δ_H_ 4.35 (1H, t, *J* = 5.6 Hz). Moreover, the ^13^C-NMR spectral analysis of **1** revealed the presence of four quaternary carbon atoms comprising one thiocarbonyl carbon (C=S, C-2) (δ_C_ 162.7); two aromatic carbons C-2′′ and C-2′′′ (δ_C_ 137.1) and one olefinic carbon (δ_C_ 131.7).

**Figure 2:**
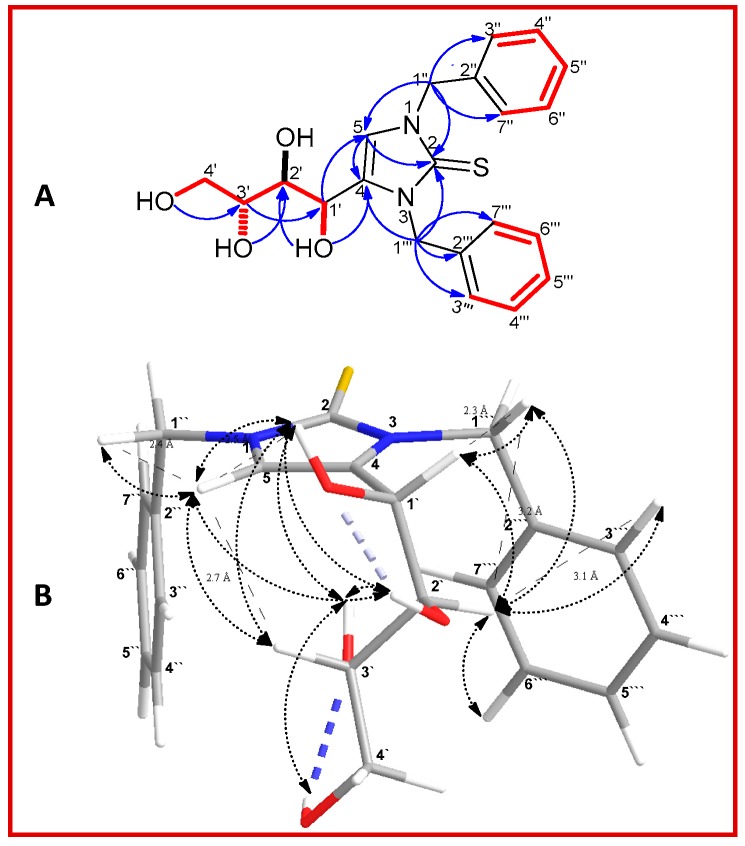
(**A**) Observed COSY (red bold bonds) and HMBC (H → C, blue) correlations of **1**. (**B**) Key NOESY (→, black) correlations and global energy minimum of **1**.

The combined analysis of the ^1^H-^1^H COSY and ^1^H-^13^C HMBC of **1** (Figure 2A) confirmed the presence tetrahydroxy butyl moiety, two symmetrical benzyl groups in addition to imidazole-2-thione central core. Key HMBC correlation from H-1′ to C-5 (δ_C_ 115.8) and OH-1′ to C-4 (δ_C_ 131.7) establish the position of the tertahydroxy butyl group at C-4, whilst HMBC correlations from H-1′′ and H-1′′′ to the thiocarbonyl carbon C-2 accompanied with correlations from H-1′′ to C-5 and H-1′′′ to C-4 confirmed the symmetrical connection of the dibenzyl groups to N-1 and N-3. Furthermore, key HMBC correlations from H-5 to C-2 and C-4 established the imidazole-2-thione central core. The relative configuration depicted in **1** was deduced from 2D NOESY spectral data. The NOESY spectrum of **1** showed that OH-1′, OH-2′ and H-3′ were oriented syn with regard to H-5 based on mutual NOE correlations (Figure 2B). In a similar manner, H-1′ and H-2′ were found to be oriented syn with regard to H-1’′ based on mutual NOE correlations. From the above data, the complete planar structure of compound **1** was established as 1,3-dibenzyl-4-[(1R*,2R*,3S*)-1,2,3,4-tetrahydroxybutyl]-1*H*-imidazole-2(3*H*)-thione.

Compounds **2** and **3** were isolated for the first time from this family. Compound **2** was isolated as pale yellow residue. In TLC, it gave an orange red spot on spraying with Dragendorff’s reagent suggesting it is a nitrogenous compound. The HR-EI mass spectrum indicated molecular ion peak at *m/z* 194.8755 and most stable fragment was the tropelium ion at *m/z* 91 indicating presence of benzyl moieties. The compound was isolated as racemic mixture of two isomers *E* and *Z*. The proton signals at δ_H_ 7.36 (2H, d, *J* = 7.5 Hz, H-5, H-9), δ_H_ 7.33(2H, d, *J* = 7.5 Hz, H-6, H-8) and δ_H_ 7.31(1H, m, H-7) indicated mono-substituted benzene ring. Two methylene protons at δ_H_ 4.78 (2H, d, *J* = 5.7 Hz, *E*-isomer) and at δ_H_ 4.46 (2H, d, *J* = 5.8 Hz, *Z*-isomer) were assigned to H-3 and another two methylene protons at δ_H_ 4.53 (2H, q, *J* = 7.2 Hz) and δ_H_ 4.58 (2H, q, *J* = 7.2 Hz) were assigned to H-1′ E isomer and *Z* isomer, respectively. The terminal methyl appeared as triplet at δ_H_ 1.36 and 1.33 assigned for H-2′ *Z* and *E* isomers, respectively. The ^13^C-NMR showed thiocarbamic carbon (C=S) at δ_C_ 190.73 (*E*) and 190.00 (*Z*) and five aromatic carbons at δ_C_ 127.96, δ_C_ 128.82, δ_C_ 127.71, δ_C_ 128.82, δ_C_ 127.96 and quaternary carbon at δ_C_ 136.96. Benzyl CH_2_ appeared at δ_C_ 49.23 (*E*), 47.27 (*Z*) and oxygenated CH_2_ at δ_C_ 66.61 (*Z*), 68.17 (*E*). Positions of the thiocarbamic carbon (C=S), the benzyl CH_2_ and oxygenated CH_2_ determined by HMBC experiment. Two and three bond correlations were observed from the two methylene protons to the thiocarbamic carbon (C=S) and two and three correlations from the benzyl CH_2_ protons to the aromatic carbons. The data compared with those reported [18] confirming the structure of compound **2** as benzyl-thiocarbamic acid-*O*-ethyl ester.

Compound **3** was isolated as a white amorphous powder. ESI-HR mass determined its molecular formula as C_23_H_39_NO by molecular ion peak at *m/z* = 346.3144. The proton signals at δ_H_ 7.17 (2H, d, *J* = 8.0 Hz) and δ_H_ 7.33 (3H, m, overlapped) indicated mono-substituted benzene ring. Doublet proton signals at δ_H_ 4.45 assigned to benzyl CH_2_ protons at H-1′. Proton signals appeared as triplet at δ_H_ 2.20 assigned to H-2 adjacent to the carbonyl carbon and at δ_H_ 1.65 as multiplet assigned to H-3. The long chain of methylene protons detected by signals at δ_H_ 1.16–1.29 with high integration number. The terminal methyl appeared at δ_H_ 0.88 as triplet. ^13^C-NMR confirmed presence of carbonyl carbon which appeared at δ_C_ 173.0. The benzyl carbon appeared at δ_C_ 43.62, while the carbon adjacent to carbonyl at δ_C_ 36.88. Presence of mono-substituted benzene ring was confirmed by five carbon signals from δ_C_ 127.55 to δ_C_ 138.40. The long chain carbons appeared at δ_C_ 22.72 to δ_C_ 31.95, while terminal methyl signal was at δ_C_ 14.16. HMBC cross peaks confirmed the linkage between the hexadecanoic acid and the benzyl amide. The data compared with those reported [19] confirming the structure as hexadecanoic acid benzyl amide. 

The known structures of **4**–**6** were identified by a comparison of their spectroscopic data with those reported in the literatures [13,14] as benzylisothiocyanate, *N*-benzyl-2-phenylacetamide and benzylurea.

### 2.2. Antioxidant Assays

In-vitro antioxidant activities for the fractions and isolates were evaluated in terms of hydrogen donating or radical scavenging ability using different radicals, such as the stable radical DPPH (1,1-diphenyl-2-picryl hydrazyl), superoxide anion, and nitric oxide with ascorbic acid as a positive control [20,21,22,23,24]. The results of DDPH radical scavenging assay showed that the total alcohol extract exhibited weak antioxidant activity with IC_50_ value of 327.7 µg/mL, while the alkaloid-rich and non-basic fractions exhibited stronger activities with IC_50_ values of 126.3 and 60 µg/mL, respectively. In superoxide radical scavenging assay, the results indicated that moderate activity was shown for petroleum ether fraction with IC_50_ equals 60 µg/mL, while the alkaloid-rich, the total alcohol extract and non-basic fractions exhibited stronger activities with IC_50_ values of 36, 20, and 15.6 µg/mL, respectively. On the other hand, the total alcohol extract showed a potent antioxidant activity in nitric oxide radical scavenging assay with IC_50_ value of 12 µg/mL even stronger than the positive control, ascorbic acid whose IC_50_ value was 28 µg/mL. In this assay, non-basic and the alkaloid-rich fractions showed moderate activities with IC_50_ values of 48 and 50 µg/mL, respectively. Petroleum ether fraction showed the weakest activity in nitric oxide assay with IC_50_ equals 201.4 µg/mL.

Among isolated compounds (**1**–**6**), the strongest antioxidant activity in the three assays was displayed by compound **1** comparable to the positive control (Table 2, Table 3, Table 4 and Table 5). Compound **1** could be a potential antioxidant compound with strong radicals scavenging activity.

## 3. Discussion

Great efforts have been made in the phytochemical and biological study of different extracts and secondary metabolites from *Salvadora persica* L. Previous studies have led to isolation of wide variety of secondary metabolites, such as alkaloids, flavonoids, saponins, and sterols, in addition to elemental sulphur and chlorides. Many important biological activities, such as antibacterial, antiviral, analgesic, anti-inflammatory, and anti-diabetic were reported [4,5,6,7,8,9,10,11,12,13]. In our study, the dried alcohol extract was subjected to acid base treatment for alkaloids extraction, followed by phytochemical separation techniques for all fractions not only the alkaloid-rich faction to find new compounds from this well-studied plant. As a result, a new sulphur-containing imidazoline alkaloid “persicaline” (**1**) along with five known compounds (**2**–**6**) are identified. 

Compounds (**2**, **3**) were reported for the first time from the family *Salvadoraeceae,* while compounds (**5**, **6**) were isolated for the first time from the roots of this species. Previous studies have revealed the presence of alkaloids and other nitrogenous compounds with varying structural patterns, including salvadorine, salvadourea, and benzyl isothiocyanate. 

However, the nitrogenous compound (**1**) obtained in our present study represents a novel type that has never been reported from the secondary metabolites of this plant. The mechanism of antioxidant activity of compound **1** is supposed to be oxidative desulfurization of 2-imidazolethiones, which is ascribed to the formation of 2-imidazolesulfinc acids, highly instable intermediates, which rapidly and irreversibly decompose into the corresponding imidazoles and sulfurous acid. The reaction produces also sulfurous acid, another effective reducing agent, which could counteract the cell oxidative stress by further conversion into sulfate [25].

**Figure 3:**
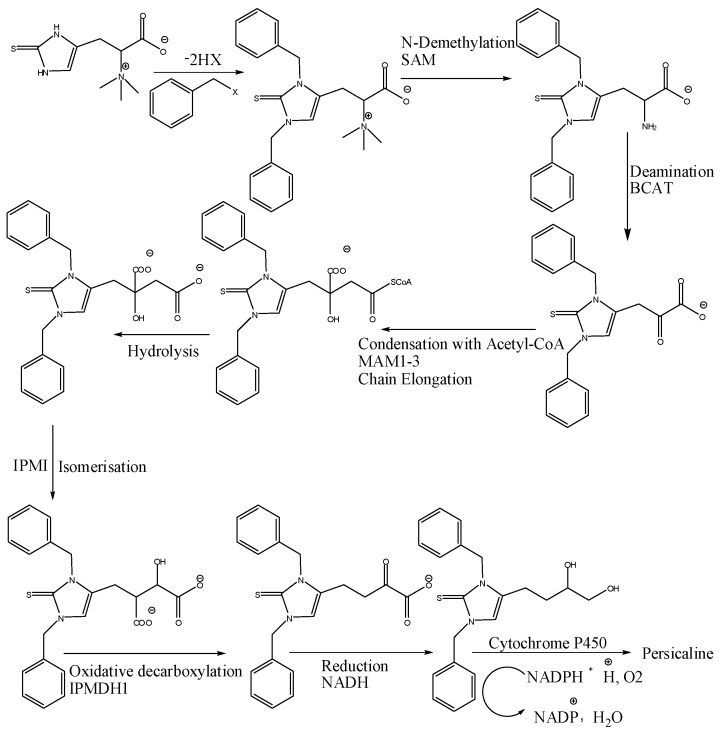
A plausible biosynthetic pathway of compound **1**.

Imidazole-2-thiones are weak organic bases protonated in acids and also considered as NH acids [16], so it was isolated from the non-basic fraction in which 0.2 M HCl (dilute acid) was used in acid-base extraction, and therefore compound **1** is considered as a genuine natural product and not an artefact from the extraction or purification processes. The biosynthetic pathway is also a proof for being a genuine natural product. It is well reported that l-histidine amino acid is the main precursor for imidazole alkaloids. The parent structure is a histidine betaine derivative with a tautomeric thiol/thione group at C-2 of the imidazole ring, which was initially isolated in 1909 from ergot and has been detected in various organisms, including plants, fungi, bacteria, animals, and humans. However, it is believed that plants obtain it from the soil, may be from a symbiotic fungus. A plausible biosynthetic pathway of **1** is proposed on the basis of a metabolic post-modification of the parent structure (Figure 3). Then the plant supposed to undergo alkylation may be with alkyl halides [26]. The quaternary amine in the aliphatic side chain was subjected to demethylation with SAM (*S*-adenosylmethionine). Plants can modify the length of this type of aliphatic side chains by several steps, as reported in [27]. In *S. persica*, we think that oxidative decarboxylation followed by oxidation with help of cytochrome P450 happened after side chain elongation process to produce our new antioxidant compound with proposed name, persicaline. 

Imidazole-2-thione derivatives are widely used in medicine as drugs or synthons for drugs. During many years, mercazolyl (1-methylimidazole-2-thione) has been used for the treatment of thyroid gland disorders [16]. Some imidazole-2-thione based drugs have therapeutic action against arthritis. Alkylthioimidazoles and their salts are used in the form of pills and capsules as dopamine-β-hydroxylase inhibitors. They also show hypotensive, diuretic, and cardiotonic effects and used for curing of ulcers and Parkinson’s disease [16]. 

Moreover, Imidazole-2-thione C-nucleosides are synthetic precursors of azido-nucleosides and fluro-nucleosides, which have anti-AIDS activity [28]. So, it is highly recommended for future screening of persicaline for different biological activities.

## 4. Materials and Methods 

### 4.1. General Experimental Procedures

IR spectra were recorded on a Nicolet 5700 FT-IR Microscope spectrometer (FT-IR Microscope Transmission, company, Waltham, MA, USA,). Optical rotations were measured on a Perkin-Elmer Model 343 polarimeter. NMR spectra were obtained on a Bruker Avance DRX700 spectrometer, company, Fallanden, Switzerland). HR-EI-MS were obtained with measurements were obtained on a Bruker microTOF mass spectrometer**.** Lichroprep RP-18 gel (40–63 m, Merck, Darmstade, Germany), Silica gel (200–300 mesh and 300–400 mesh), and Silica gel H (Qingdao Oceanic Chemical Co., Qingdao, China) were used for column chromatography. TLC pre-coated silica-gel 60 F_254_ glass-backed plates (0.25 mm, ALUGRAM^®^ SIL G/UV254, Macherey-Nagel, Germany) and RP-18 F_254s_ plates (0.25 mm, Merck, Germany) were used in the screening and isolation process. Fractions were monitored by TLC and spots were visualized by spraying the sheets with p-anisaldehyde/H_2_SO_4_ reagent followed by heating at 110 °C for 1–2 min. All of the solvents and chemicals used were of analytical reagent grade (GPR™, BDH limited, Poole, UK), and water was doubly distilled before use.

### 4.2. Plant Material

The roots of *S. persica* L. were collected from the medicinal plants garden of the Pharmacognosy department, College of Pharmacy, King Saud University, Riyadh, Saudi Arabia in spring 2015. The plant was authenticated by Dr. Mohammed Yusuf, Department of Pharmacognosy, College of Pharmacy, KSU. Voucher specimens (No. 15616) were deposited at the herbarium of Pharmacognosy Department, College of Pharmacy, KSU, Riyadh, Saudi Arabia.

### 4.3. Extraction, Fraction, and Isolation

**Figure 4:**
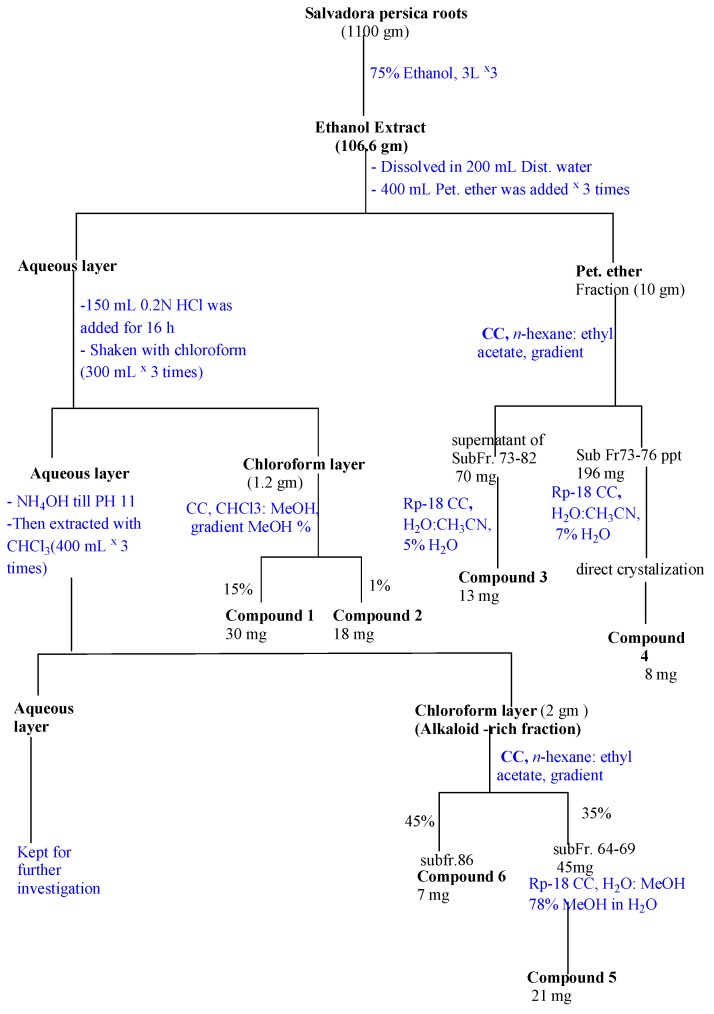
Extraction, fractionation and purification scheme for *S. persica* roots.

The air-dried roots of S. persica (1.1 kg) were exhaustively extracted by cold maceration in 75% aqueous ethanol at room temperature. The total alcohol extract was concentrated under reduced pressure to yield a brown residue (106.6 g, 9.7%). The dried alcoholic extract was subjected to solvent fractionation and acid base treatment for alkaloid extraction [15] (Figure 4). Solvent fractionation was done using petroleum ether and water. The petroleum ether layer was subjected to silica gel column chromatography using *n*-Hexane-Ethyl acetate in gradient elution manner to give two promising fractions that further subjected to reversed phase columns using water-acetonitrile in gradient manner to give compounds **3** and **4**. The aqueous layer was stirred with 0.2 N HCl for 16 h then was shaken with chloroform to remove the non-basic material. The chloroform fraction was subjected to silica gel column using CHCl_3_–MeOH gradient to give compounds **1** and **2**. The aqueous layer was basified with ammonium hydroxide until pH 11, and then was extracted with chloroform. The chloroform phase was evaporated to obtain the Alkaloid Rich Fraction (ARF). ARF was subjected to silica gel column using *n*-Hexane-Ethyl acetate gradient to give two promising fractions; one of them was subjected to reversed phase column using water-Methanol in gradient manner to give compound **5**, while the other was subjected to direct crystallization to give compound **6**.

### 4.4. Antioxidant Assay

#### 4.4.1. DPPH Scavenging Radical Assay

In-vitro antioxidant screening for the extract, fractions and compounds was done in terms of hydrogen donating or radical scavenging ability using the stable radical DPPH (1,1-diphenyl-2-picrylhydrazyl) by similar method to that reported [20,21,22]. A stock solution of each sample was prepared to contain 1 mg/mL and then diluted to final concentrations of (6.25, 12.5, 25, 50, 100) µg/mL, in methanol. 500 µL of (80 µg/mL solution) DPPH was added to 500 µL of each sample different concentrations. A positive control (ascorbic acid) was prepared in the same way as samples, while the blank solution by adding 500 µL methanol to 500 µL of DPPH (80 µg/mL solution). As DPPH is sensitive to light, all of the test solutions left to react at room temperature in darkness for at least 30 min. The spectrophotometer was set at 517 nm and the absorbance was adjusted at zero for methanol. The absorbance of blank, positive control, and samples were recorded. The disappearance of DPPH was recorded and the percent inhibition of the DPPH radical by samples and positive control was calculated as follows:

% Inhibition or % radical scavenging activity = (Ab − As)/Ab × 100(1)

where Ab is the absorbance of blank (has the highest value) and As is the absorbance of sample or positive control (ascorbic acid).

The tests were done in triplicate. The IC_50_ (the concentration of sample required to rummage half of DPPH radicals) values were computed by linear regression, where the abscissa signs to the concentration of the tested samples and the ordinate the average percent of scavenging capacity. One way ANOVA test was used to judge the statistical significance and level was set to 0.05. Values of all the parameters are viewed as mean ± SD of three independent measurements.

#### 4.4.2. Superoxide Anion Scavenging Radical Assay

Reactive oxygen species (ROS), such as superoxide anions and other free radicals are formed during metabolism and specialized physiological reactions. Repeated exposure to these radicals is considered a main cause of aging, neurodegenerative, and inflammatory diseases due to gradual damage of cellular components, such as DNA and proteins [23]. 

The superoxide anion radical scavenging activities of the extracts and isolates were assessed using the method described by Fontana et al [23] with slight modification. To various concentrations of the samples (6.25–100 µg/mL), 1.0 mL of phosphate buffer (0.1 M, pH 7.2), 1.0 mL of NADH (2 mM), 1.0 mL of NBT (0.5 mM), and 0.1 mL of PMS (0.03 mM) were added. After 5 min incubation at ambient temperature, the absorbance was read at 562 nm against a reagent blank to detect the quantity of formazan generated. The standard used was ascorbic acid. All of the tests were performed in triplicate. The % scavenging/inhibition were calculated as below

% scavenging/inhibition = [(Absorbance _Control-_ Absorbance _Test)_/Absorbance _control_] × 100 (2)

where A_control_ = absorbance of control sample and A_test_ = absorbance in the presence of extracts, compounds or standard.

#### 4.4.3. Nitric Oxide Scavenging Radical Assay

Nitric oxide is classified as a free radical because of its unpaired electron and important reactivity with certain types of proteins and other free radicals, such as superoxide in vivo. NO is synthesized in the vascular endothelial cells, certain neuronal cells, and phagocytes. Chronic exposure to nitric oxide radical can cause various carcinomas and inflammatory conditions [24]. 

In vitro quenching of NO radical is one of the methods that can be used to measure antioxidant activity in which nitric oxide is generated from sodium nitroprusside interaction with oxygen to produce nitrite ions, which were measured by the Griess reaction. The procedure done was reported by Nagmoti et al with slight modifications [24]. Three milliliters of 10 mM sodium nitroprusside in phosphate buffered saline (pH 7.4) were added to different concentrations of (6.25–100 µg/mL) tested samples. After 60 min incubation at 25 °C, the resulting solution was then added to 5.0 mL of Griess reagent (1% sulphanilamide, 0.1% NEDD in 2% H_3_PO_4_). At 546 nm, the absorbance of the chromophore formed was measured against a reagent blank. Percentage inhibition of the nitrite ions generated was observed. Ascorbic acid was used as a standard for comparison. The free radical scavenging activity was determined by computing % inhibition as above.

## 5. Conclusions

A new sulphur-containing imidazoline alkaloid, persicaline (**1**), along with five known compounds (**2**–**6**) was identified. Compounds (**2**, **3**) were reported for the first time from the family *Salvadoraeceae*. The antioxidant activities of the fractions and isolates were evaluated using in vitro DPPH, superoxide anion, and nitric oxide radicals scavenging assays. Compound (**1**) showed reasonable antioxidant activity comparable to ascorbic acid in the three done assays.

The nitrogenous compound (**1**) obtained in our present study represents a novel type that has never been reported from the secondary metabolites of this plant. Many Drugs or synthons for drugs from imidazole-2-thione derivatives are widely used in medicine, so, it is highly recommended for future screening of persicaline for different biological activities, especially as antitumor due to its reasonable antioxidant activity. Also, future investigation for isolation and characterization of bioactive compounds from *S. persica* for formulation of new bio-products and/or more potent drugs is highly recommended.

## Figures and Tables

**Table 1:** ^1^H (700 MHz) and ^13^C-NMR (175 MHz) data of compound **1** in DMSO-*d*_6_ (***δ*** in ppm, *J* in Hz).

Position	Compound 1	
	δ_H_	δ_C_
1		
2		162.7 C
3		
4		131.7 C
5	7.08, s	115.8 CH
1′	4.62, m	62.9 CH
2′	3.38, m	71.9 CH
3′	3.40, m	70.7 CH
4′	a 3.53, m	63.3 CH_2_
	b 3.37, m	
1′′	a 5.32, (d, 15.9)	49.9 CH_2_
	b 5.23, (d, 15.9)	
2′′		137.1 C
3′′	7.36, (d, 7.5)	127.9 CH
4′′	7.31, (d, 7.5)	128.4 CH
5′′	7.31, m	127.7 CH
6′′	7.31, (d, 7.5)	128.4 CH
7′′	7.36, (d, 7.5)	127.9 CH
1′′′	5.42, s	47.6 CH_2_
2′′′		137.1 C
3′′′	7.22, (d, 7.5)	126.8 CH
4′′′	7.37, (d, 7.5)	128.6 CH
5′′′	7.25, m	127.2 CH
6′′′	7.37, (d, 7.5)	128.6 CH
7′′′	7.22, (d, 7.5)	126.8 CH
OH-1′	5.11, (d, 8.1)	
OH-2′	4.68, (d, 8.1)	
OH-3′	4.55, (d, 8.1)	
OH-4′	4.35, (t, 5.6)	

**Table 2:** % Inhibition of DPPH radical of different concentrations of important fractions and active compound (**1**).

Sample (µg/mL)	% Inhibition ± SD			
	Total Alcohol Extract	Non-Basic Fraction	Compound 1	Ascorbic Acid
6.25	1 ± 0.2	10.2 ± 0.95	8.8 ± 0.82	40.32 ± 0.05
12.5	2.4 ± 0.03	27.91 ± 8.83	13.68 ± 1.91	94.22 ± 0.04
25	4 ± 0.01	31.12 ± 1.7	25.07 ± 0.89	96.56 ± 0.04
50	7.5 ± 0.01	46.1 ± 16.69	61.30 ± 13.48	96.86 ± 0.02
100	15.5 ± 0.14	71.94 ± 0.34	60.65 ± 1.46	96.76 ± 0.02

The statistical significance was calculated from one way ANOVA analysis and level was set to 0.05. Values of all parameters are expressed as mean ± SD of three independent measurements.

**Table 3:** % Inhibition of superoxide anion radical of different concentrations of tested fractions and compounds.

Sample (µg/mL)	% Inhibition ± SD										
	Total Alcohol Extract	Pet. Ether Fraction	Non Basic Fraction	Alkaloid-Rich Fraction	Isolated Compounds						Ascorbic Acid
					1	2	3	4	5	6	
6.25	9.56 ± 3.09	6.49 ± 3.02	29.61 ± 7.77	12.93 ± 6.73	21.74 ± 14.25	5.99 ± 2.86	-	13.13 ± 3.75	0.46 ± 0.80	3.50 ± 2.35	57.10 ± 6.51
12.5	33.00 ± 11.85	16.16 ± 3.74	47.35 ± 7.31	27.63 ± 10.78	32.89 ± 11.41	10.02 ± 2.14	-	34.55 ± 13.91	4.00 ± 3.19	9.03 ± 1.56	66.16 ± 6.64
25	56.50 ± 6.17	26.20 ± 1.65	62.20 ± 3.67	40.83 ± 10.00	51.41 ± 13.73	19.19 ± 3.37	-	44.00 ± 7.01	4.50 ± 3.05	17.03 ± 4.36	75.36 ± 6.98
50	75.83 ± 9.04	46.80 ± 8.02	72.13 ± 2.62	55.90 ± 0.63	58.80 ± 15.63	32.80 ± 4.24	-	57.17 ± 7.49	6.50 ± 4.65	22.13 ± 3.69	84.36 ± 5.49
100	86.96 ± 4.99	61.47 ± 10.60	82.33 ± 1.33	66.16 ± 5.31	72.30 ± 5.32	42.47 ± 6.32	-	71.26 ± 6.42	7.76 ± 4.31	26.19 ± 2.70	88.40 ± 5.04

The statistical significance was calculated from one way ANOVA analysis and level was set to 0.05. Values of all parameters are expressed as mean ± SD of three independent measurements. - No inhibitory effect observed.

**Table 4:** % Inhibition of nitric oxide radical of different concentrations of tested fractions and compounds.

Sample (µg/mL)	% Inhibition ± SD										
	Total Alcohol Extract	Pet. Ether Fraction	Non Basic Fraction	Alkaloid-Rich Fraction	Isolated Compounds						Ascorbic Acid
					1	2	3	4	5	6	
6.25	31.43 ± 13.05	5.16 ± 2.48	7.40 ± 4.56	10.56 ± 5.34	17.86 ± 15.85	10.40 ± 6.32	3.68 ± 2.57	6.66 ± 2.10	0.72 ± 1.25	7.40 ± 4.56	23.86 ± 8.91
12.5	51.43 ± 3.14	10.43 ± 3.25	20.06 ± 8.58	23.09 ± 3.04	28.10 ± 22.28	14.16 ± 4.35	10.30 ± 2.62	11.26 ± 0.28	1.46 ± 1.26	12.63 ± 5.43	41.43 ± 7.77
25	63.69 ± 10.70	19.36 ± 8.72	36.71 ± 16.53	33.50 ± 8.52	42.40 ± 23.65	25.61 ± 5.18	10.28 ± 1.22	19.20 ± 6.40	3.66 ± 1.25	19.43 ± 6.21	48.16 ± 7.16
50	71.60 ± 10.48	21.63 ± 10.28	50.50 ± 10.21	50.03 ± 4.00	59.16 ± 14.84	30.03 ± 3.35	11.73 ± 3.21	21.86 ± 5.35	5.13 ± 1.20	28.46 ± 12.0	66.03 ± 8.24
100	76.83 ± 5.01	27.00 ± 7.85	57.00 ± 10.21	56.54 ± 6.03	70.36 ± 14.73	33.06 ± 1.86	14.68 ± 5.02	25.61 ± 5.18	6.61 ± 0.08	38.30 ± 5.63	77.36 ± 4.22

The statistical significance was calculated from one way ANOVA analysis and level was set to 0.05. Values of all parameters are expressed as mean ± SD of three independent measurements.

**Table 5:** Radicals scavenging IC_50_ values of fractions and isolated compounds in the three assays (**1−6**).

Compound/Fraction	IC_50_ µg/mL (µM) in DPPH Assay	IC_50_ µg/mL (µM) in Superoxide Anion Assay	IC_50_ µg/mL (µM) in Nitric Oxide Assay
Total alcohol extract	327.7	20	12
Pet. Ether fraction	399.1	60	201.4
Non-basic fraction	60.0	15.6	48
Alkaloid-rich fraction	126.3	36	50
1	38.5 (0.1)	32.7(0.08)	34.5 (0.09)
2	1551.4 (7.9)	46.5 (0.24)	161.0 (0.82)
3	1659.4 (4.8)	Inactive	502.3 (1.45)
4	666.2 (4.5)	36 (0.24)	223.9 (1.5)
5	Inactive	757.5 (3.36)	809.6 (3.59)
6	1180 (7.9)	196.5 (1.31)	130.2 (0.87)
Ascorbic Acid	7.0 (0.04)	1.5 (0.01)	28 (0.16)

## Supplementary Materials

Supplementary materials are available online, ^1^H-, ^13^C-NMR, DEPT, COSY, HSQC, HMBC, and NOESY spectra of compound **1** and ^1^H- and ^13^C-NMR data of compounds **2**, **3.**
