# Supplementary material for: Persicaline, A New Antioxidant Sulphur-Containing Imidazoline Alkaloid from Salvadora persica Roots
Source: Molecules. 2018 Feb 23;23(2):483. doi: 10.3390/molecules23020483 (PMC6017864; doi:10.3390/molecules23020483)
Supplement: Supplementary file 1 [file molecules-23-00483-s001.pdf]

SUPPLEMENTARY MATERIAL

Persicaline, a new antioxidant sulphur-containing imidazoline alkaloid from *Salvadora persica* roots

Mohamed F. Abd El Halim[a], Wael M Abdel-Mageed[a,b,*], Omer A. Basudan[a] and Ali A. El-Gamal[a,c,*]

[a]*Pharmacognosy Department, King Saud University, College of Pharmacy, , P.O. Box 2457, Riyadh 11451, Saudi Arabia e-mails:* wabdelmageed@ksu.edu.sa, & *aelgamal00@yahoo.com*

[b] *Pharmacognosy Department, Assiut University, Faculty of Pharmacy, , Assiut P.O. Box 71526, Egypt*

[c]*Pharmacognosy Department, Mansoura University, Faculty of Pharmacy, , Mansoura P.O. Box 35516, Egypt*

Abstract

Salvadora persica L. is a popular chewing stick commonly known as 'miswak'. During our ongoing research activities on the chemical constituents of *Salvadora persica* roots, a new sulphur-containing imidazoline alkaloid 1,3-Dibenzyl-4-(1,2,3,4-tetrahydroxy-butyl)-1,3-dihydro-imidazole-2-thione, persicaline, (**1**) along with five known compounds (**2-6**) are identified. Compounds (**2-3**) were reported for the first time from the family *Salvadoraeceae*. The structure of the new compound was established by extensive spectroscopic data and HR-MS. The antioxidant activities of the fractions and isolates were evaluated using different *in vitro* methods such as DPPH, superoxide anion and nitric oxide radicals scavenging assays. Compound (**1**) showed a promising antioxidant activity with IC_{50} 0.1, 0.08 and 0.09 μM in the three assays respectively comparable to ascorbic acid.

Keywords: *Salvadora persica*; *Persicaline*; Imidazoline Alkaloids; Sulphur-containing compounds; Radical Scavenging Activity.

Content

Table S1 ^{1}H NMR and ^{13}C NMR spectroscopic data of compounds **2**

Table S2 ^{1}H NMR and ^{13}C NMR spectroscopic data of compounds **3**

Figure S1. ^{1}H NMR spectrum of compound (**1**) (700 MHz, DMSO-d_6)
Figure S2. ^{13}C NMR spectrum of compound (**1**) (175 MHz, DMSO-d_6)
Figure S3. DEPT 135 spectrum of compound (**1**) (175 MHz, DMSO-d_6)
Figure S4. ^{1}H-^{1}H COSY spectrum of compound (**1**) (700 MHz, DMSO-d_6)
Figure S5. ^{1}H-^{13}C HSQC spectrum of compound (**1**) (700 MHz, DMSO-d_6)
Figure S6. ^{1}H-^{13}C HMBC spectrum of compound (**1**) (700 MHz, DMSO-d_6)
Figure S7. ^{1}H-^{1}H NOESY spectrum of compound (**1**) (700 MHz, DMSO-d_6)
Figure S8. Key NOESY (→, black) correlations and global energy minimum of **1**

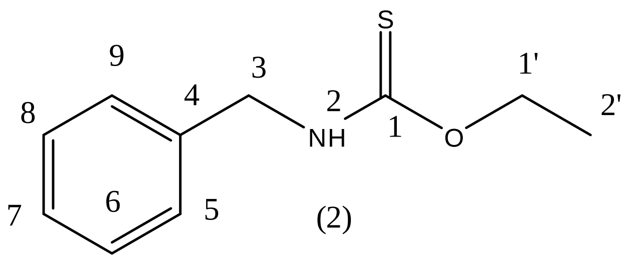

(2)

Benzyl-thiocarbamic acid *O*-ethyl ester

Table S1 ^{1}H (500 MHz) of compound **2** and ^{13}C (125 MHz) NMR spectral data of compounds **2** in $CDCl_3$

**Position**	**H^{1} NMR data**<br>**δ_H (ppm) (*J* in Hz)**	**C^{13} NMR data**<br>**δ_C (ppm)**
**1**	**-**	190 (*Z*)<br>190.73 (*E*)
**2**	6.5 br. (*Z*)<br>6.9 br. **(***E***)**	**-**
**3**	4.46 (2H, d, *J* = 5.8 Hz, CH_2, *Z*)<br>4.78 (2H, d, *J* = 5.7 Hz, CH_2, *E*)	47.27 (*Z*)<br>49.31 (*E*)
**4**	-	136.63<br>136.96
**5**	7.36 (1H, d, *J*= 7.5Hz)	128.92
**6**	7.33(1H, d, *J*= 7.5Hz)	127.98
**7**	7.31(1H, m)	127.79
**8**	7.33(1H, d, *J*= 7.5Hz)	127.98
**9**	7.36 (1H, d, *J*= 7.5Hz)	128.92
**1'**	4.58 (2H, q, *J* = 7.2 Hz, *Z*)<br>4.53 (2H, q, *J* = 7.2 Hz, *E*),	66.69(*Z*)<br>68.17 (*E*)
**2'**	1.37 (3H, t, *J* = 7.2 Hz, *Z*)<br>1.33 (3H, t, *J* = 7.2 Hz, *E*)	14.32 (*Z*)<br>14.38 (*E*)

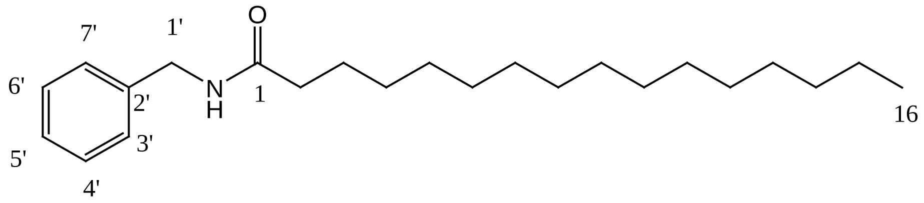

Table S2 ^{1}H (500 MHz) of compound **2** and ^{13}C (125 MHz) NMR spectral data of compounds **3** in $CDCl_3$

**Position**	**H^1 NMR data** **δ_H (ppm) (*J* in Hz)**	**C^{13} NMR data** **δ_C (ppm)**
1	-	173.0
2	2.20 (t)	36.88
3	1.65 (m)	22.72 to 31.95
4	1.16-1.29	22.72 to 31.95
5	1.16-1.29	22.72 to 31.95
6	1.16-1.29	22.72 to 31.95
7	1.16-1.29	22.72 to 31.95
8	1.16-1.29	22.72 to 31.95
9	1.16-1.29	22.72 to 31.95
10	1.16-1.29	22.72 to 31.95
11	1.16-1.29	22.72 to 31.95
12	1.16-1.29	22.72 to 31.95
13	1.16-1.29	22.72 to 31.95
14	1.16-1.29	22.72 to 31.95
15	1.16-1.29	22.72
16	0.88	14.16.
1'	4.45 (d)	43.62
2'	-	138.40
3'	7.17 (1H, d, *J*= 8.0 Hz)	127.9
4'	7.33(1H, m, overlapped)	128.8
5'	7.33(1H, m, overlapped)	127.55
6'	7.33(1H, m, overlapped)	128.8
7'	7.17 (1H, d, *J*= 8.0 Hz)	127.9

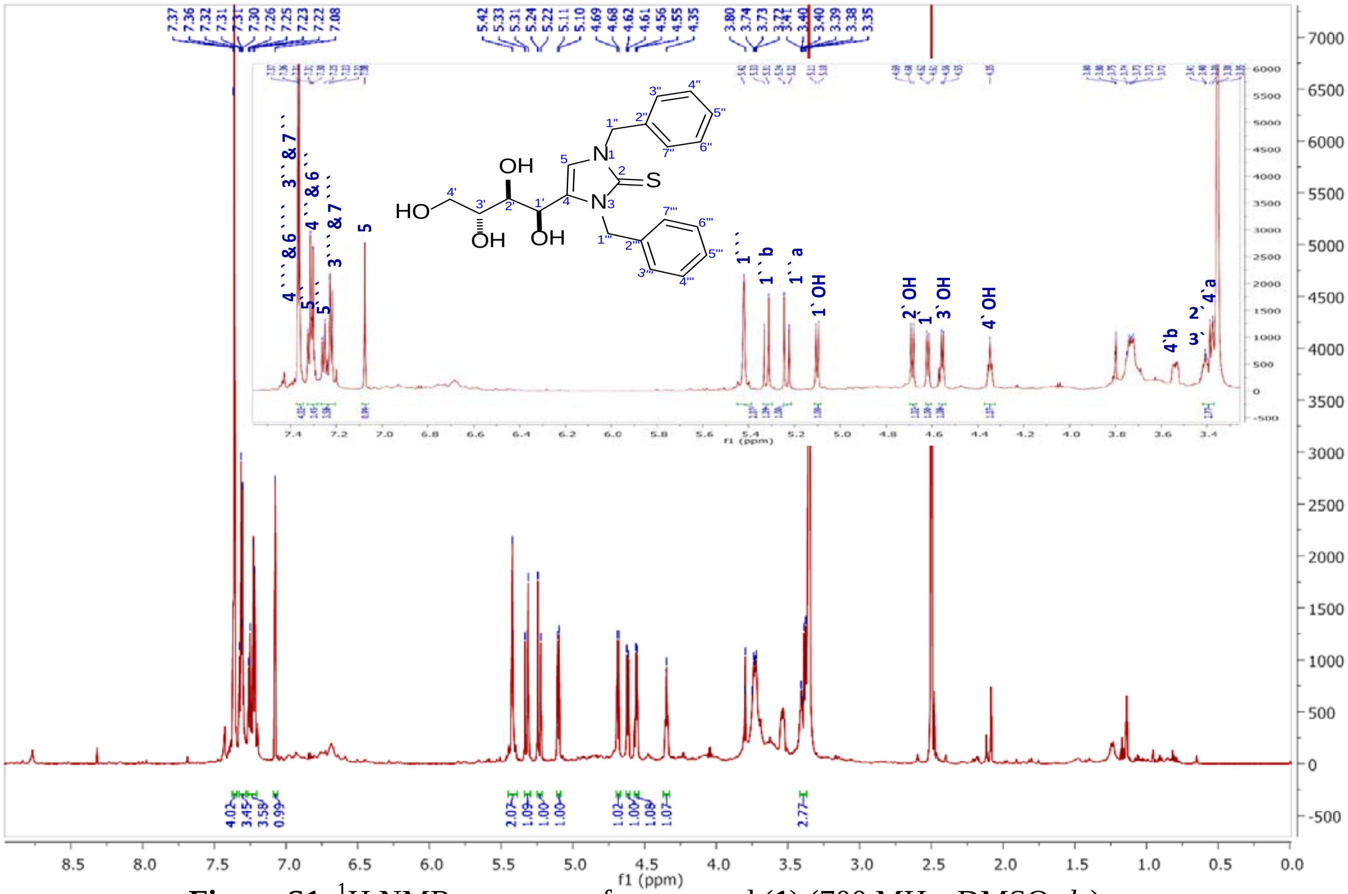

Figure S1. ^{1}H NMR spectrum of compound (**1**) (700 MHz, DMSO-d_6)

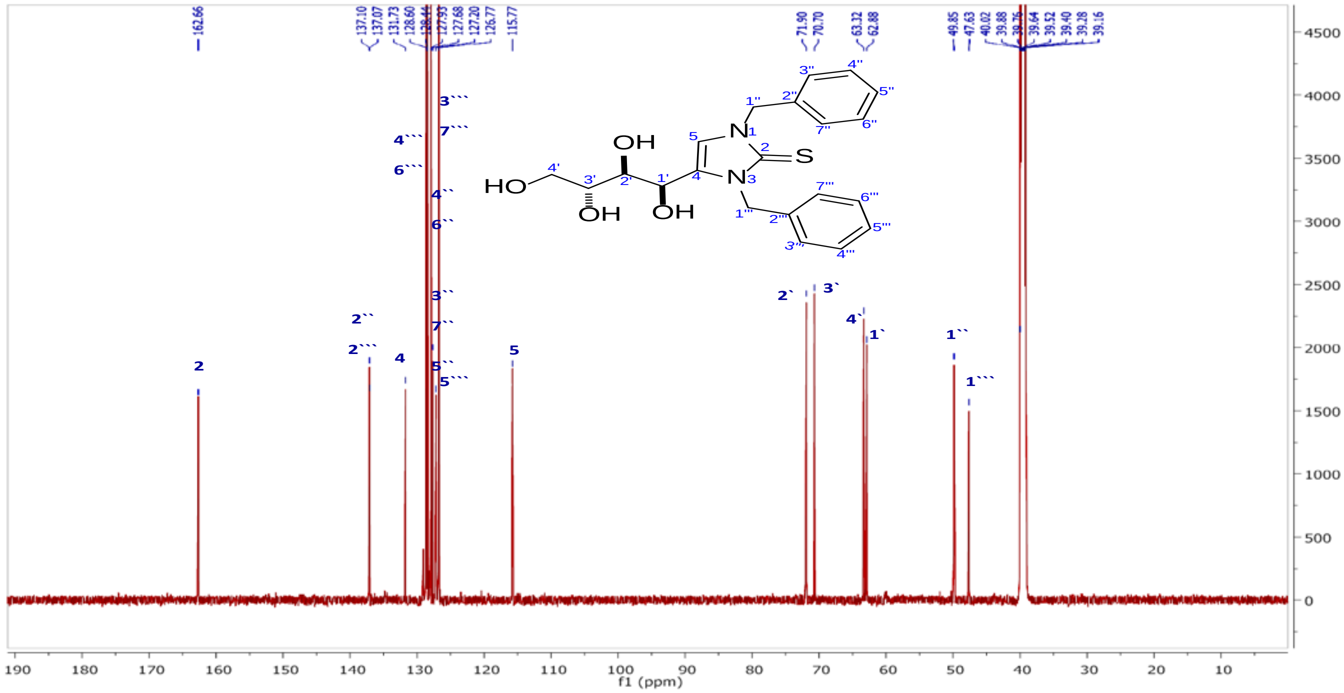

Figure S2. ^{13}C NMR spectrum of compound (**1**) (175 MHz, DMSO-d_6)

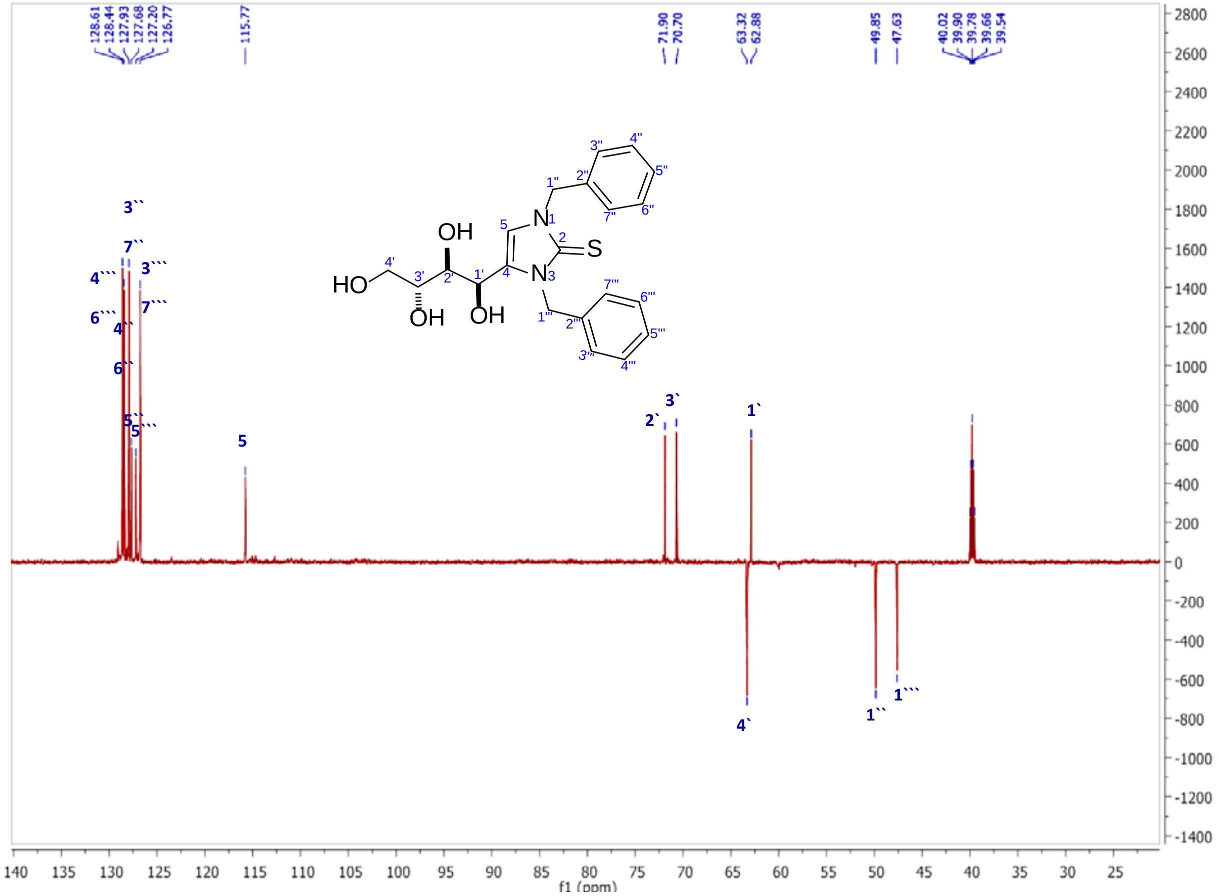

Figure S3. DEPT 135 spectrum of compound (**1**) (175 MHz, DMSO-d_6)

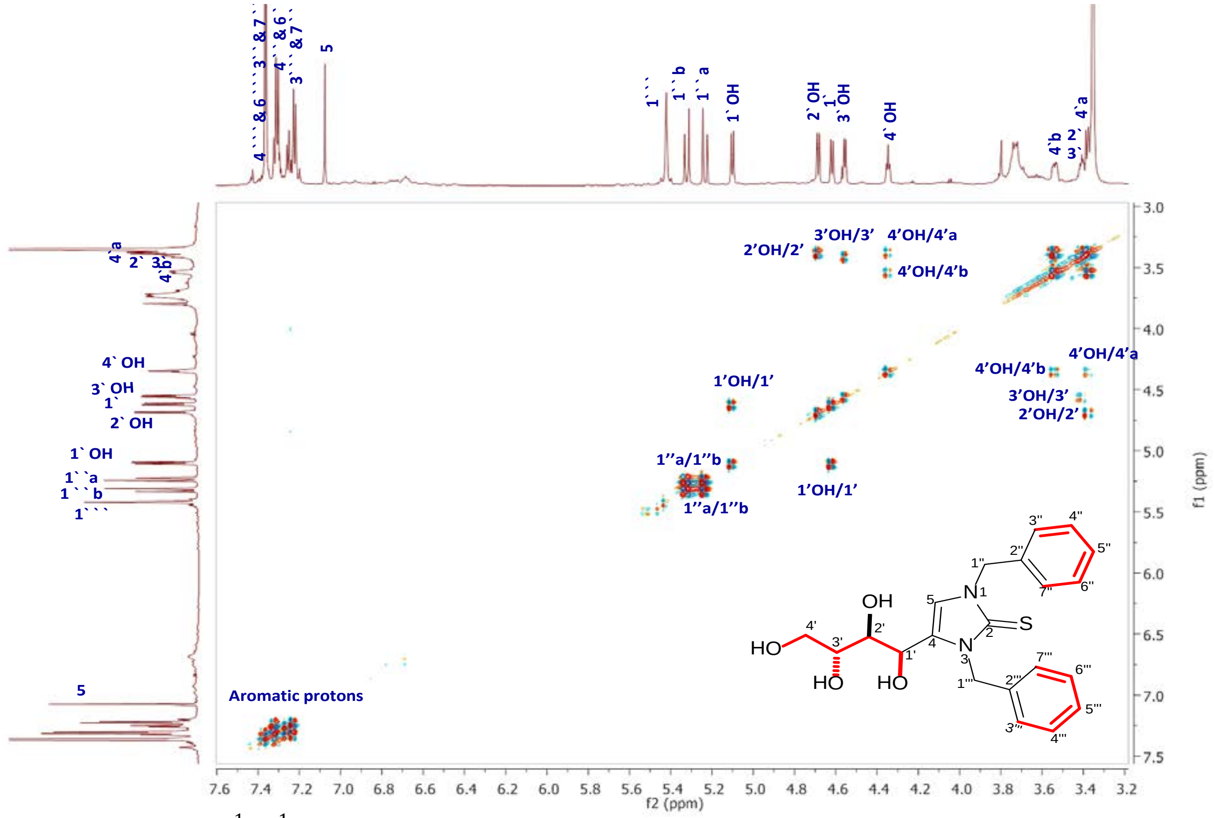

Figure S4. ^{1}H-^{1}H COSY spectrum of compound (**1**) (700 MHz, DMSO-d_6)

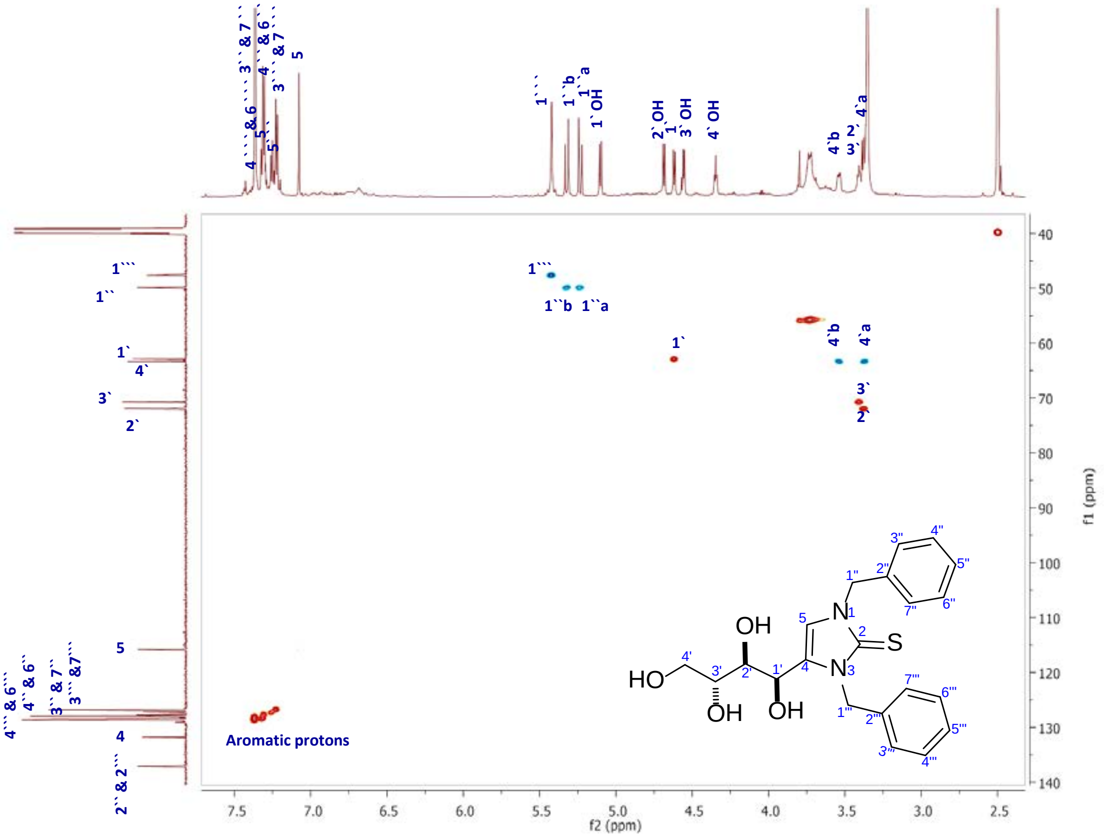

Figure S5. ^{1}H-^{13}C HSQC spectrum of compound (**1**) (700 MHz, DMSO-d_6)

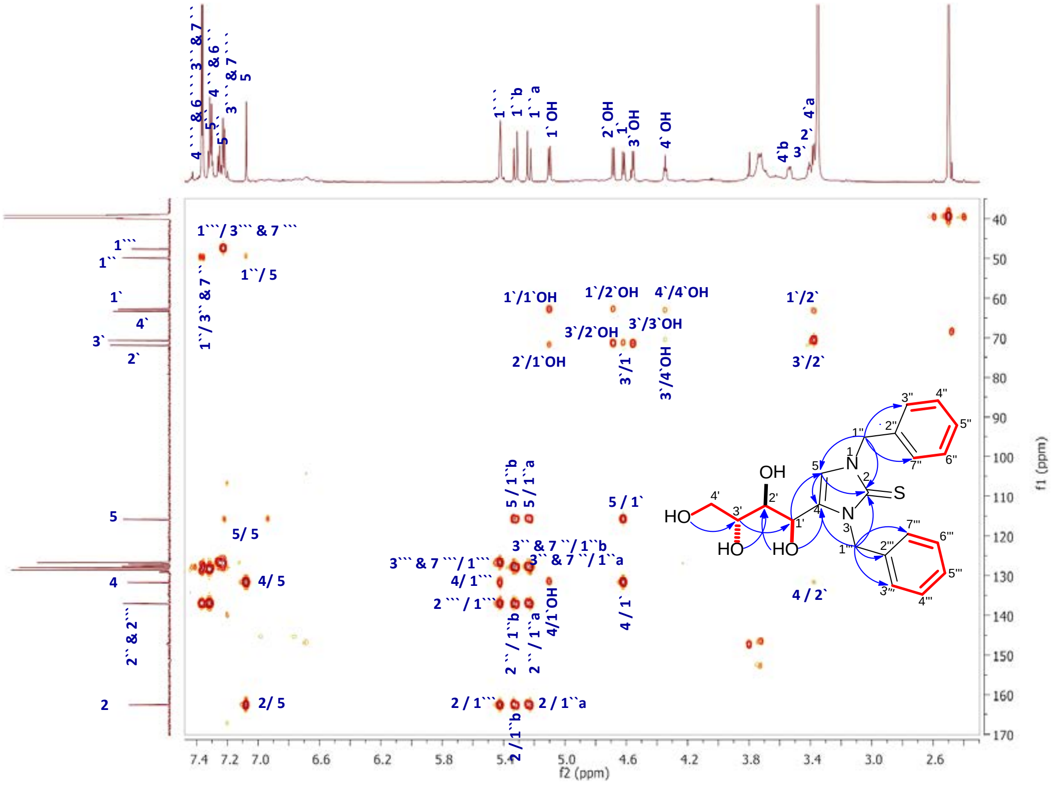

Figure S6. ^{1}H-^{13}C HMBC spectrum of compound (**1**) (700 MHz, DMSO-d_6)

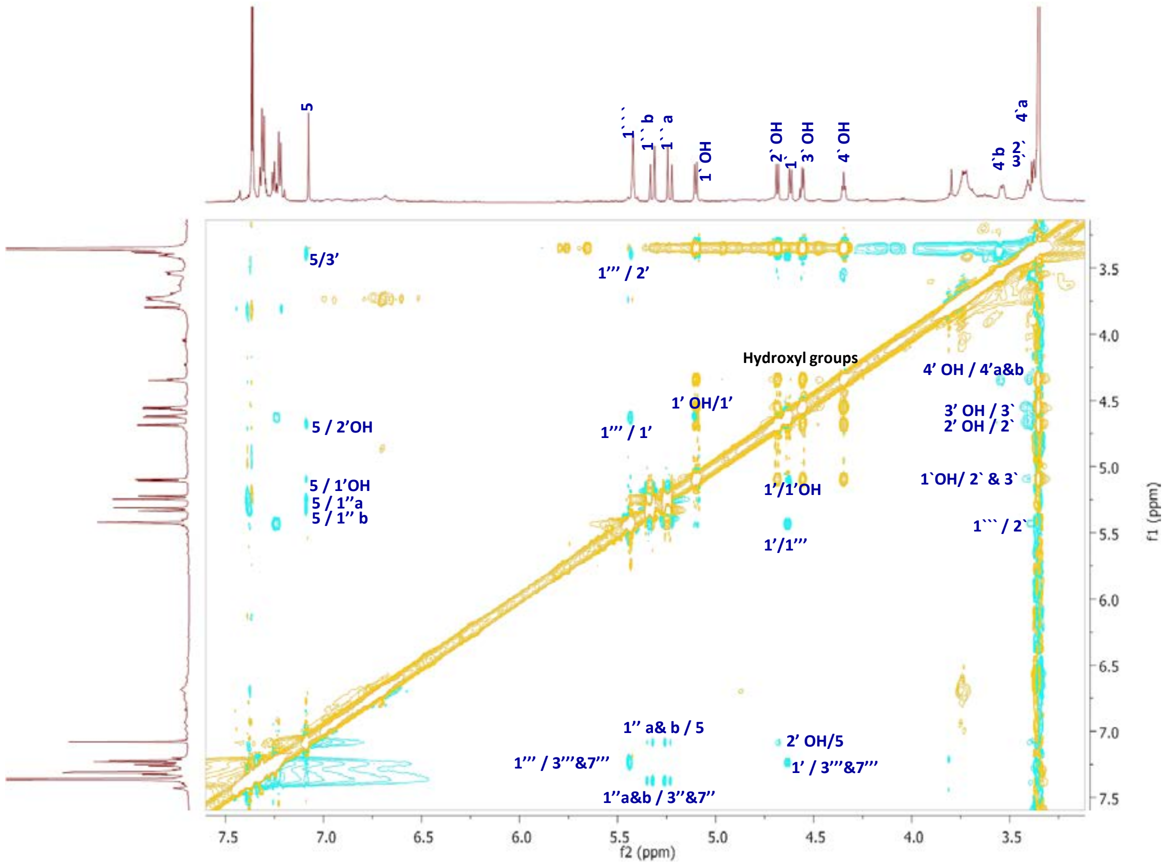

Figure S7. ^{1}H-^{1}H NOESY spectrum of compound (**1**) (700 MHz, DMSO-d_6)

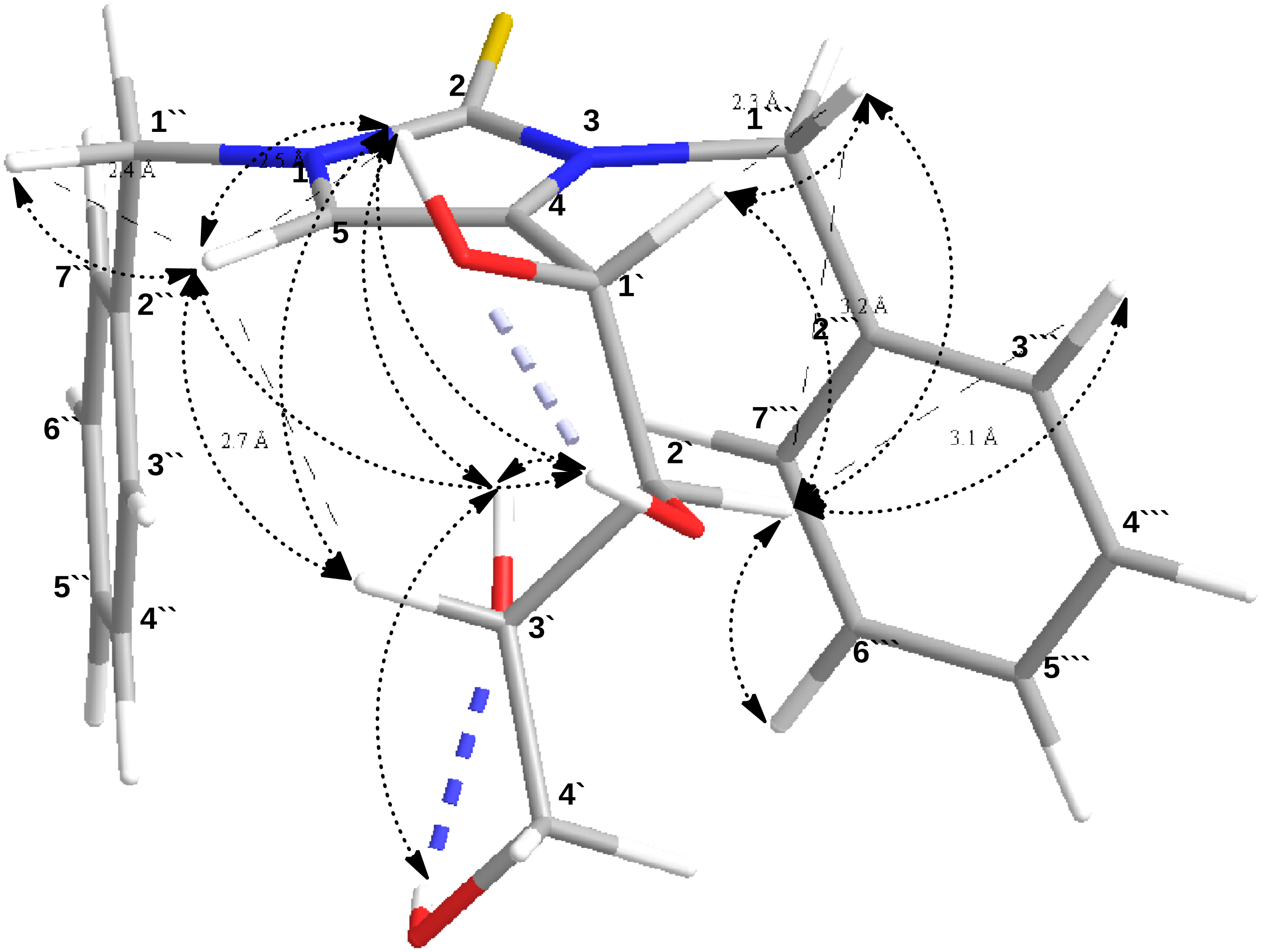

Figure S8. Key NOESY (→, black) correlations and global energy minimum of **1**.
